# Author Correction: Zinc homeostasis governed by Golgi-resident ZnT family members regulates ERp44-mediated proteostasis at the ER-Golgi interface

**DOI:** 10.1038/s41467-023-39273-z

**Published:** 2023-06-12

**Authors:** Yuta Amagai, Momo Yamada, Toshiyuki Kowada, Tomomi Watanabe, Yuyin Du, Rong Liu, Satoshi Naramoto, Satoshi Watanabe, Junko Kyozuka, Tiziana Anelli, Tiziana Tempio, Roberto Sitia, Shin Mizukami, Kenji Inaba

**Affiliations:** 1 Institute of Multidisciplinary Research for Advanced Materials, Tohoku University, Sendai, Miyagi 980-8577 Japan; 2 Department of Chemistry, Graduate School of Science, Tohoku University, Sendai, Miyagi 980-8578 Japan; 3 Department of Molecular and Chemical Life Sciences, Graduate School of Life Sciences, Tohoku University, Sendai, Miyagi 980-8577 Japan; 4 Department of Chemistry, Faculty of Science, Tohoku University, 6-3 Aramaki-aza-Aoba, Aoba-ku, Sendai, Miyagi 980-8578 Japan; 5 Department of Ecological Developmental Adaptability Life Sciences, Graduate School of Life Sciences, Tohoku University, Sendai, Miyagi 980-8577 Japan; 6 Division of Genetics and Cell Biology, Vita-Salute University, IRCCS Ospedale San Raffaele, 20132 Milan, Italy; 7 Core Research for Evolutional Science and Technology (CREST), Japan Agency for Medical Research and Development (AMED), Chiyoda, Tokyo Japan; 8 Medical Institute of Bioregulation, Kyushu University, Fukuoka, 812−8582 Japan; 9 Present Address: Department of Biological Sciences, Faculty of Science, Hokkaido University, Kita 10 Nishi 8, Kita-ku, Sapporo, Japan

**Keywords:** Transport carrier, Golgi, Secretion

Correction to: *Nature Communications* 10.1038/s41467-023-38397-6, published online 9 May 2023

The original version of this article contained a duplication of Supplementary Fig. 1 in the Supplementary Information. The Supplementary Information file has been corrected.

## Supplementary information

Updated Supplementary Information

