# Supplementary material for: Zinc homeostasis governed by Golgi-resident ZnT family members regulates ERp44-mediated proteostasis at the ER-Golgi interface
Source: Nat Commun. 2023 May 9;14:2683. doi: 10.1038/s41467-023-38397-6 (PMC10170084; doi:10.1038/s41467-023-38397-6)
Supplement: Supplementary file 1 — Supplementary Information [file 41467_2023_38397_MOESM1_ESM.pdf]

**Supplementary information**

# Zinc homeostasis governed by Golgi-resident ZnT family members regulates ERp44-mediated proteostasis at the ER-Golgi interface

Yuta Amagai, Momo Yamada, Toshiyuki Kowada, Tomomi Watanabe, Yuyin Du, Rong Liu, Satoshi Naramoto, Satoshi Watanabe, Junko Kyozuka, Roberto Sitia, Shin Mizukami, Kenji Inaba*

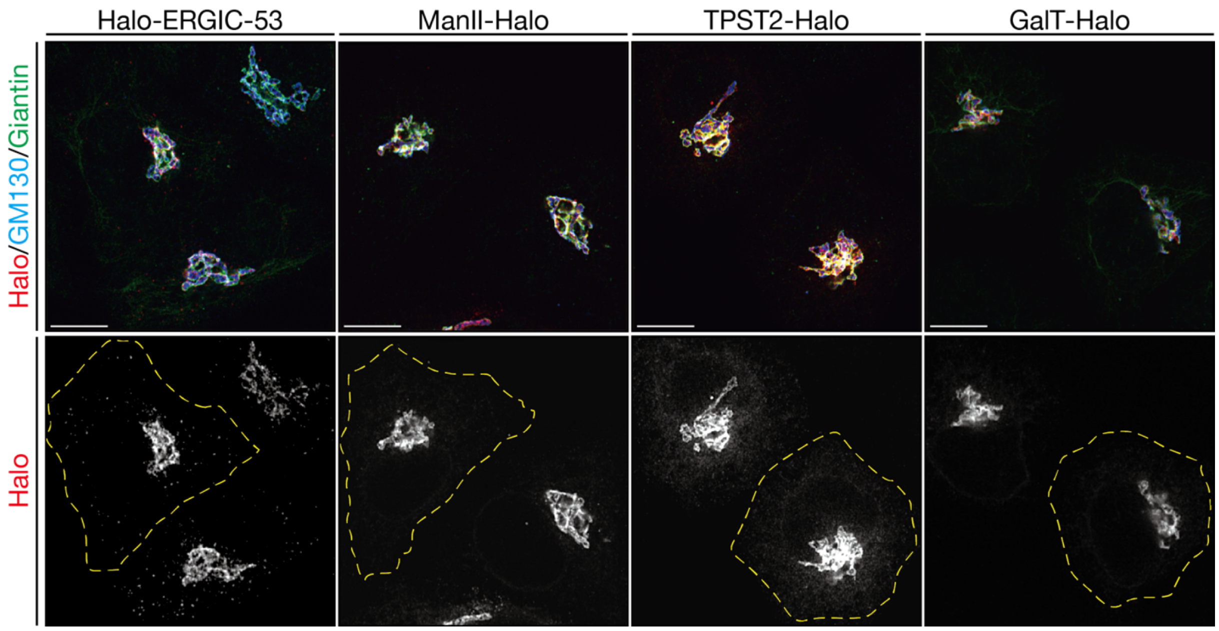


**Supplementary Figure 1. Localization of Halo-fused Golgi marker proteins**

HeLa Kyoto cells transfected with indicated Halo-tagged Golgi marker proteins were incubated with HaloTag-TMR Ligand for 30 min and fixed with 4% PFA. Cells were further immunostained for GM130 (*cis*-Golgi) and giantin (medial-Golgi). The Golgi localization of these Halo-fused proteins was confirmed by more than three independent experiments. Fluorescence images were acquired by Airyscan super-resolution microscopy (Carl Zeiss). Scale bars, 10 µm.

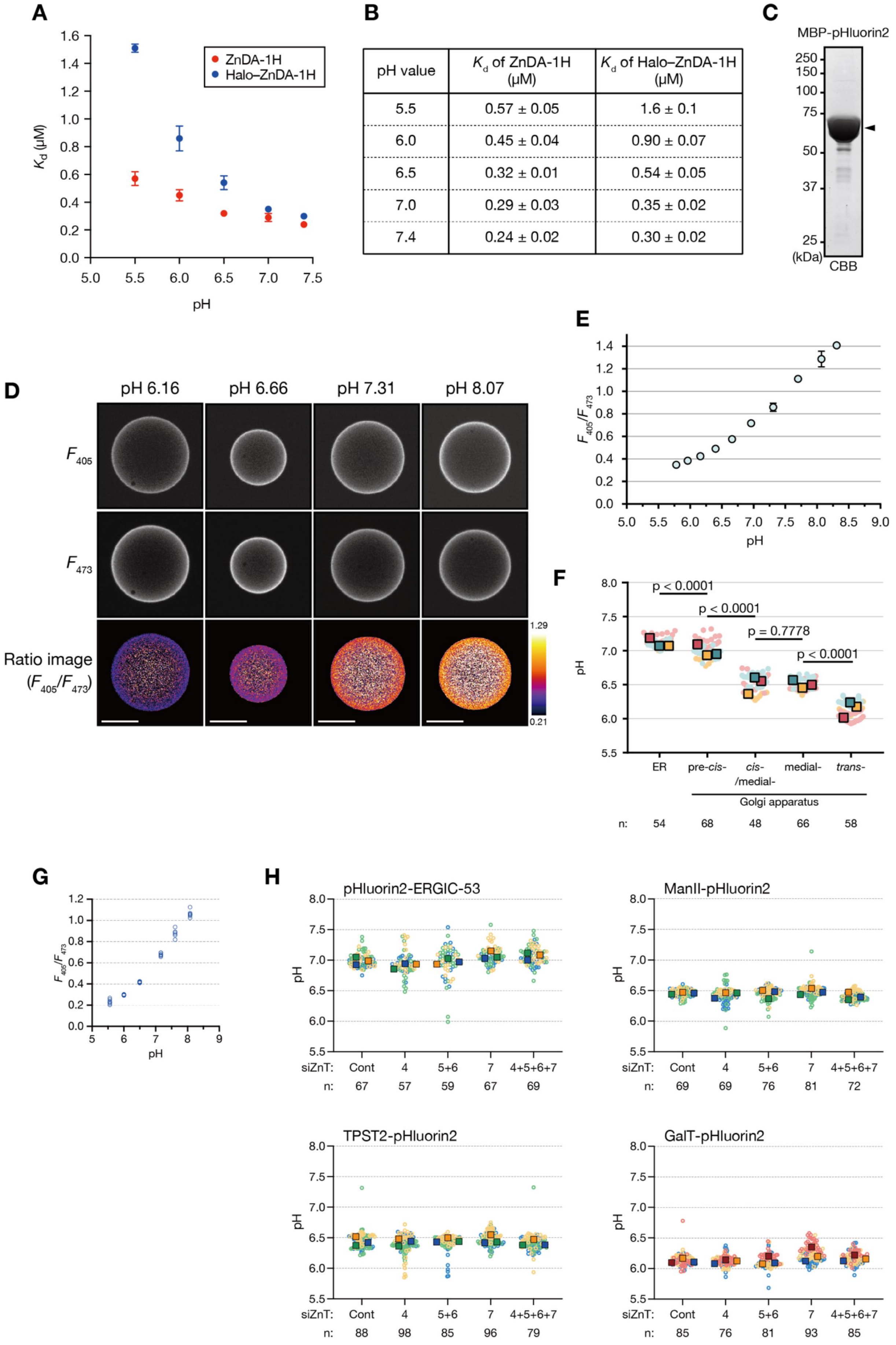


| pH value | $K_d$ of ZnDA-1H (μM) | $K_d$ of Halo–ZnDA-1H (μM) |
|---|---|---|
| 5.5 | 0.57 ± 0.05 | 1.6 ± 0.1 |
| 6.0 | 0.45 ± 0.04 | 0.90 ± 0.07 |
| 6.5 | 0.32 ± 0.01 | 0.54 ± 0.05 |
| 7.0 | 0.29 ± 0.03 | 0.35 ± 0.02 |
| 7.4 | 0.24 ± 0.02 | 0.30 ± 0.02 |

**Supplementary Figure 2. Effects of pH value on the $Zn^{2+}$-binding affinity of ZnDA-1H and effects of ZnT-silencing on pH value in the Golgi cisternae**

(A, B) $K_d$ of ZnDA-1H or Halo–ZnDA-1H at different pH values. Data are the means ± SEM.

(C) MBP-pHluorin2 proteins were bacterially expressed, affinity purified using Amylose resin, and visualized by SDS-PAGE and CBB staining. Similar results were obtained from two independent experiments.

(D) Fluorescence images of MBP-pHluorin2-immobilized Amylose bead upon excitation at 405 nm ($F_{405}$) or 473 nm ($F_{473}$) under several pH conditions. Scale bars, 50 µm.

(E) Fluorescence ratios of pHluorin2-immobilized beads in buffers between pH 5.78 and 8.31. These values were used for a calibration of (F). Data are the means ± SEM (n = 5 beads for pH 5.78–7.31 and n = 6 beads for pH 7.7–8.31).

(F) pH measurements of each subregion. HeLa Kyoto cells transfected with pHluorin2-tagged organelle markers were imaged in the imaging buffer. pHluorin2 fluorescence ratio based on the fluorescence intensities were acquired, and pH values were calculated assuming a linear relationship between pH and pHluorin2 fluorescence ratio. The sample size (n) represents the number of cells from 3 independent experiments. Circles indicate individual cells, and rectangles indicate the median of each experiment. Data were analyzed by Tukey's multiple comparison test (two-sided).

(G) Fluorescence ratios of pHluorin2-immobilized Amylose beads in buffers between pH 5.56 and 8.08. These values were used for a calibration of (H). Circles indicate individual data points (n = 7 beads for pH 5.72; n = 8 beads for pH 6.18; n = 8 beads for pH 6.68; n = 7 beads for pH 7.41; n = 6 beads for pH 7.97; n = 7 beads for pH 8.42).

(H) pH measurements of each subregion of cells transfected with siRNAs against ZnT4, ZnT5+6, ZnT7, or ZnT4+5+6+7. pH values were determined as in (F). The sample size (n) represents the number of cells from 3 independent experiments. Circles indicate individual cells, and rectangles indicate the median of each experiment. Source numerical data and unprocessed gel data are provided in the Source Data file.

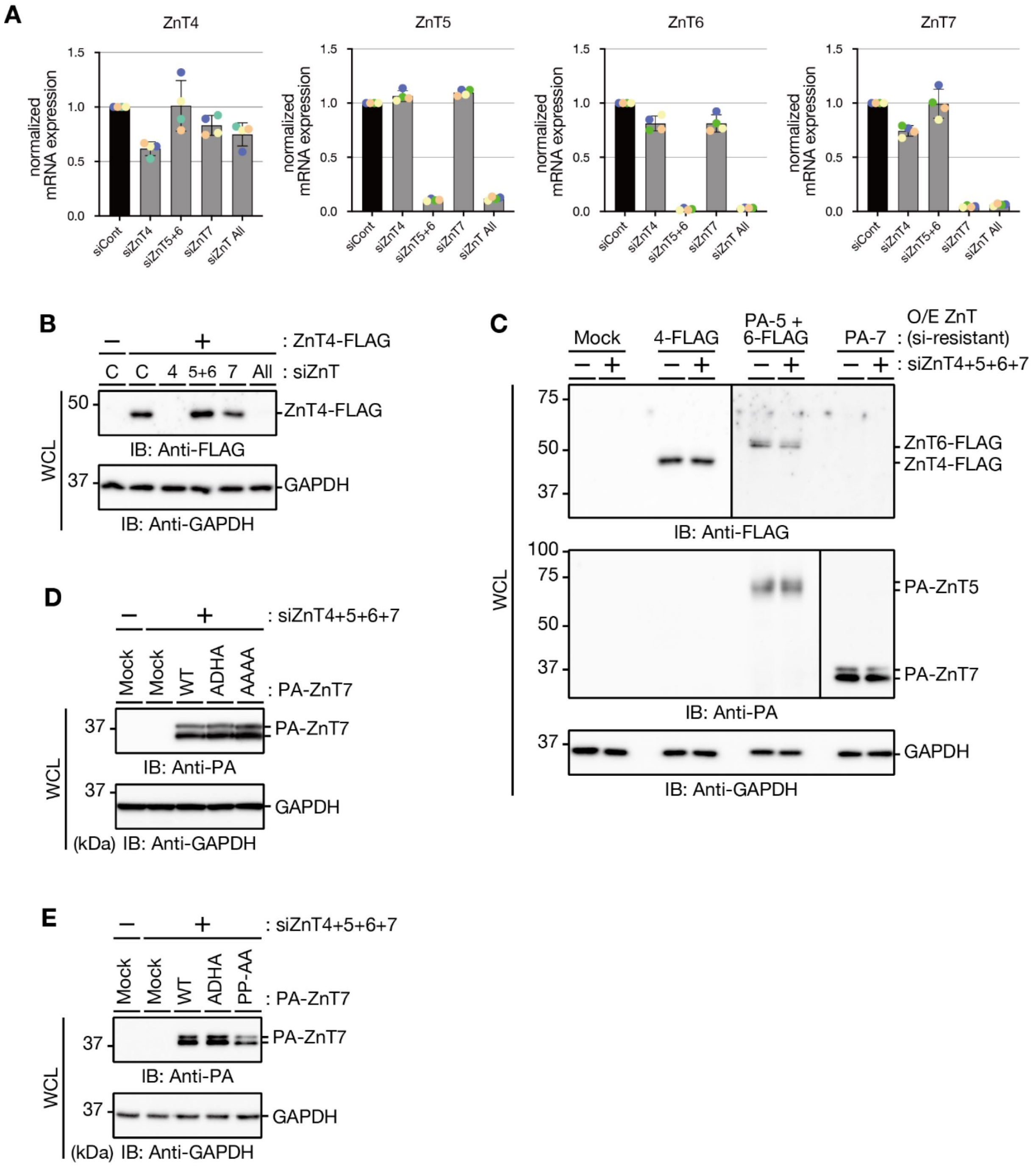


**Supplementary Figure 3. Evaluations of siRNA-mediated knockdown of ZnTs and siRNA-resistance of sir-ZnT constructs**

(A) mRNAs from HeLa Kyoto cells transfected with indicated siRNAs for 48 were extracted, and cDNAs were synthesized by RT-PCR. Normalized mRNA expressions of ZnT4, ZnT5, ZnT6, and ZnT7 were analyzed by qRT-PCR. PGK1 was used as a standard gene. Data are the means ± SD, and circles represent individual data point (N = 4 biological replicates).

(B) HeLa Kyoto cells transfected with siRNAs against ZnT4, ZnT5, ZnT6, and ZnT7 were further transfected with ZnT4-FLAG. After 48 h incubation, whole cell lysates (WCL) were

analyzed by immunoblotting against FLAG and GAPDH.

(C) ZnT4/5/6/7-silencing cells were transfected with FLAG- or PA-tagged siRNA resistant (si-resistant) ZnT4, 5+6, or 7. WCL were analyzed by immunoblotting.

(D) Protein expressions of si-resistant ZnT7(WT, ADHA, and AAAA) were confirmed by immunoblotting.

(E) Protein expressions of si-resistant ZnT7(WT, ADHA, and PP-AA) were confirmed by immunoblotting.

The siRNAs resistances of these constructs were confirmed by two independent experiments. Source numerical data and unprocessed blotting images are provided in the Source Data file.

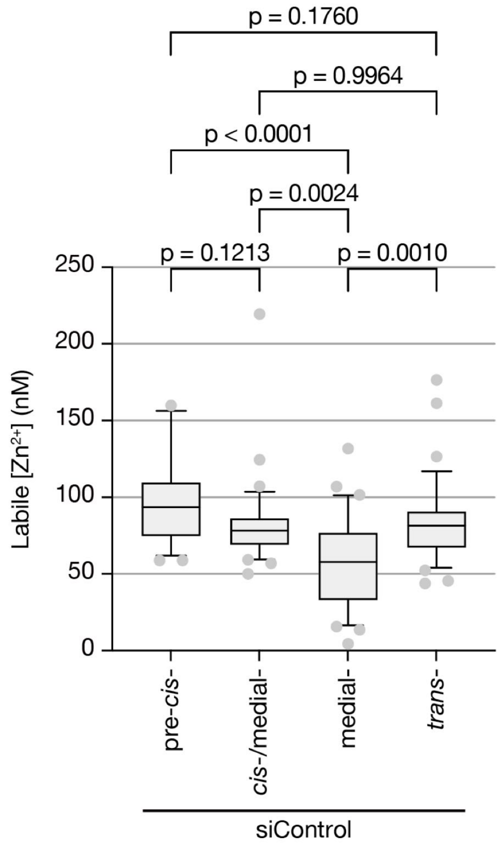


| | Labile [$Zn^{2+}$] (nM) Means ± SEM | n |
|---|---|---|
| pre-*cis*- | 100 ± 7 | 23 |
| *cis*-/medial- | 82 ± 5 | 35 |
| medial- | 57 ± 5 | 36 |
| *trans*- | 84 ± 5 | 36 |

**Supplementary Figure 4. Statistical comparison of labile $Zn^{2+}$ concentration between the different Golgi cisternae in siControl-treated cells**

The labile $Zn^{2+}$ concentrations in the different Golgi cisternae of siControl-treated HeLa Kyoto cells are shown in a box-and-whisker plot (center line, median; box limits, upper and lower quartiles; whiskers, 10–90% range; points, outliers). Data were analyzed with Tukey's multiple comparison test (two-sided). Source numerical data are provided in the Source Data file.

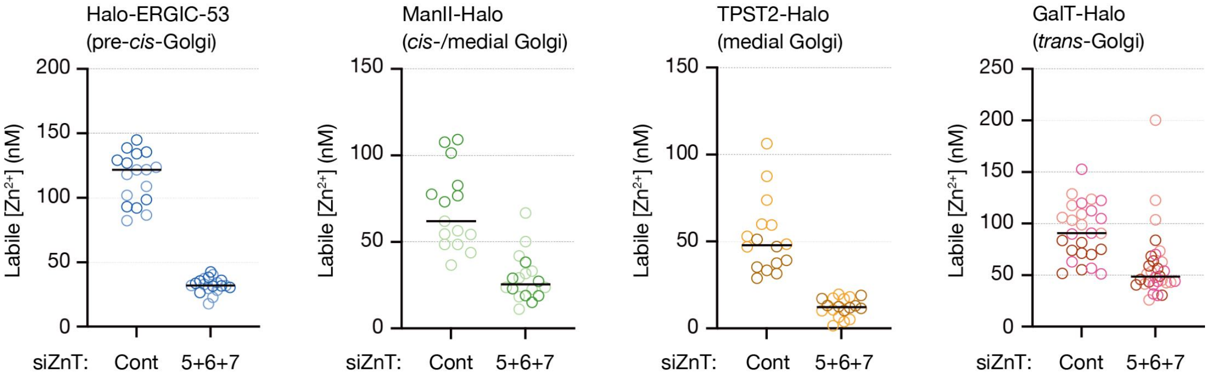


**Supplementary Figure 5. Effects of simultaneous knockdown of ZnT5/6/7 on the labile $Zn^{2+}$ at the Golgi apparatus**

HeLa Kyoto cells transfected with siRNAs against ZnT5/6/7 simultaneously were further transfected with Halo-tagged Golgi markers. Halo-tagged proteins were labeled with ZnDA-1H and HTL-TMR at a 50:1 ratio. Fluorescence images were obtained with a confocal microscopy LSM980 equipped with 63x oil-immersion lens (NA 1.40). The 440 nm and 568 nm lasers were used for excitation of ZnDA-1H and TMR, respectively. Experiments and image quantification were performed as described at Methods. Circles indicate individual data points from 2 independent experiments, and bars indicates the medians. Halo-ERGIC-53: siCont, n = 17 cells; siZnT5+6+7, n = 19 cells. ManII-Halo: siCont, n = 15 cells; siZnT5+6+7, n = 18 cells. TPST2-Halo: siCont, n = 16 cells; siZnT5+6+7, n = 18 cells. GalT-Halo: siCont, n = 26 cells; siZnT5+6+7, n = 30 cells. Source numerical data are provided in the Source Data file.

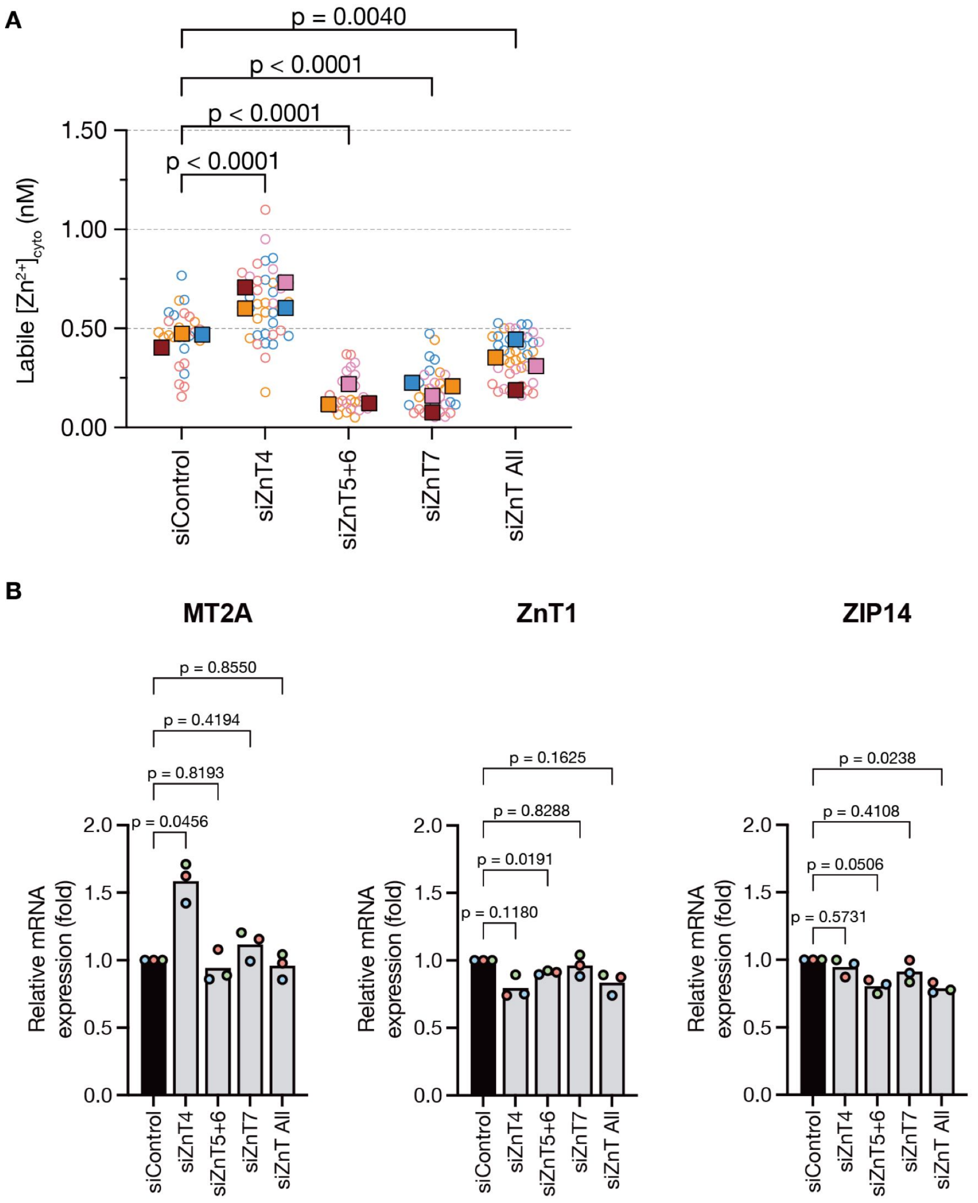


**Supplementary Figure 6. Effects of ZnTs-knockdown on the zinc homeostasis**

(A) Measurements of the cytosolic labile [$Zn^{2+}$] using ZnDA-2H. HeLa Kyoto cells were transfected with the indicated siRNAs and a Halo protein with a nuclear export signal (NES). A circular dot indicates each datapoint, and a rectangle dot indicates the median of each experiment (siControl, n = 28 cells from 3 independent experiments; siZnT4, n = 40 cells from 4 independent experiments; siZnT5+6, n = 28 cells from 3 independent experiments; siZnT7, n = 31 cells from 4 independent experiments; siZnT All, n = 42 cells from 4 independent experiments). One-way ANOVA followed by Tukey's multiple comparison test was used for statistical analysis.

(B) mRNAs from HeLa Kyoto cells transfected with indicated siRNAs for 48 were extracted, and cDNAs were synthesized by RT-PCR. Normalized mRNA expressions of MT2A, ZnT1,

and ZIP14 were analyzed by qRT-PCR. PGK1 was used as a standard gene. Bars are the means, and circles represent individual data point (N = 3 biological replicates). One-way ANOVA followed by Dunnett's multiple comparison test was used for statistical analysis.
Source numerical data are provided in the Source Data file.

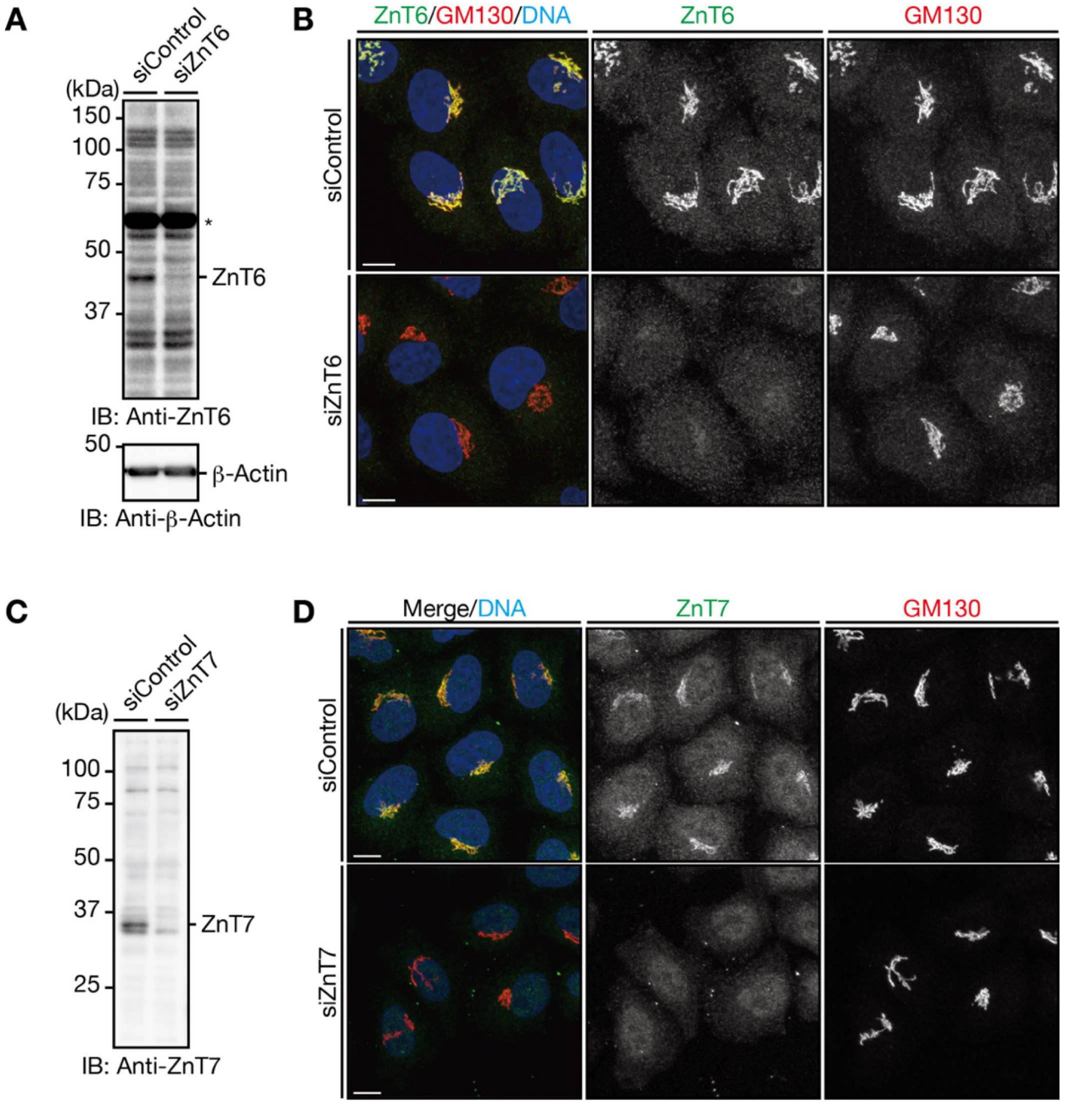


**Supplementary Figure 7. ZnT6 and ZnT7 primarily localize at the interior of Golgi cisterna**

(A) Whole cell lysates from HeLa Kyoto cells transfected with siControl or siZnT6 were analyzed by immunoblotting. Asterisk indicates non-specific band.

(B) Control or ZnT6-silencing cells were fixed and immunostained for ZnT6 and GM130. Fluorescence images were acquired by a laser scanning confocal microscopy (FV1000, Olympus). Scale bars, 10 µm.

(C) Whole cell lysates from HeLa Kyoto cells transfected with siControl or siZnT7 were analyzed by immunobotting.

(D) Control or ZnT7-silencing cells were fixed and immunostained for ZnT7 and GM130. Fluorescence images were acquired by a laser scanning confocal microscopy (FV1000, Olympus). Scale bars, 10 µm.

Similar results were obtained from two independent experiments (A–D). Unprocessed blotting images are provided in the Source Data file.

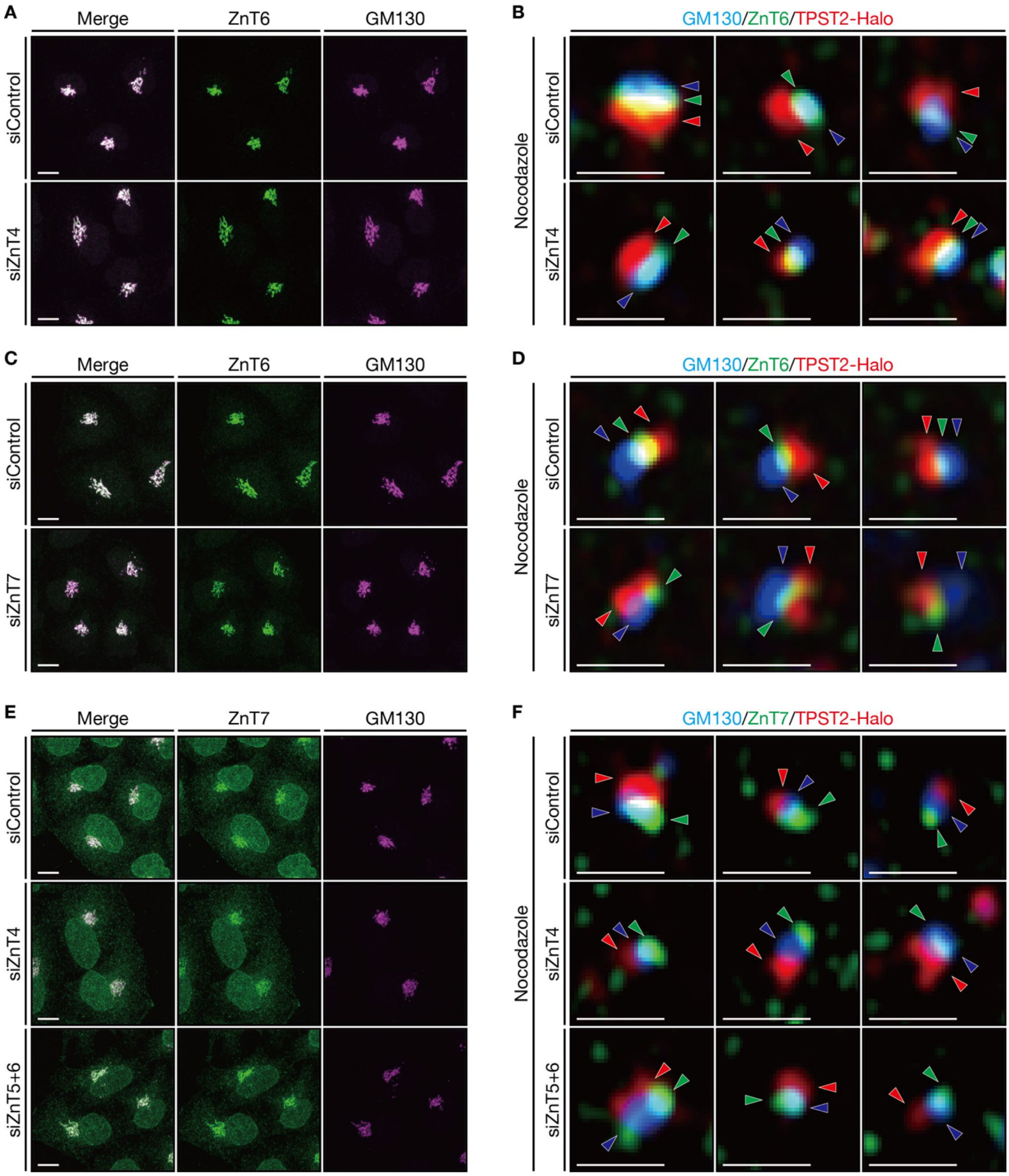


**Supplementary Figure 8. Knockdowns of the Golgi-resident ZnTs do not affect the localization of ZnT6 and ZnT7**

(A–D) Localization of ZnT6 upon ZnT4- or ZnT7-knockdown. HeLa Kyoto cells transfected with siZnT4 (A) or siZnT7 (C) were immunostained for ZnT6 and GM130. HeLa Kyoto cells transfected with TPST2-Halo and siZnT4 (B) or siZnT7 (D) were incubated with 33 μM nocodazole for 4 h and immunostained for ZnT6 and GM130. Arrowheads indicate the signals of GM130 (blue), ZnT6 (green) and TPST2-Halo (red). ZnT6 signals were observed between

GM130 and TPST2-Halo or colocalized with GM130 in all conditions.

(E–F) Localization of ZnT7 upon ZnT4- or ZnT5/6-knockdown. HeLa Kyoto cells transfected with siZnT4 or siZnT5/6 were immunostained for ZnT7 and GM130 (E). The Golgi ministacks prepared as in (B and D) were immunostained for GM130 and ZnT7. Arrowheads indicate the signals of GM130 (blue), ZnT7 (green) and TPST2-Halo (red). ZnT7 signals were observed at the upstream of GM130 or colocalized with GM130 in all conditions. Each panel shows 3 representative images. Scale bars are 10 μm for (A), (C), and (E) or 1 μm for (B), (D), and (F), respectively.

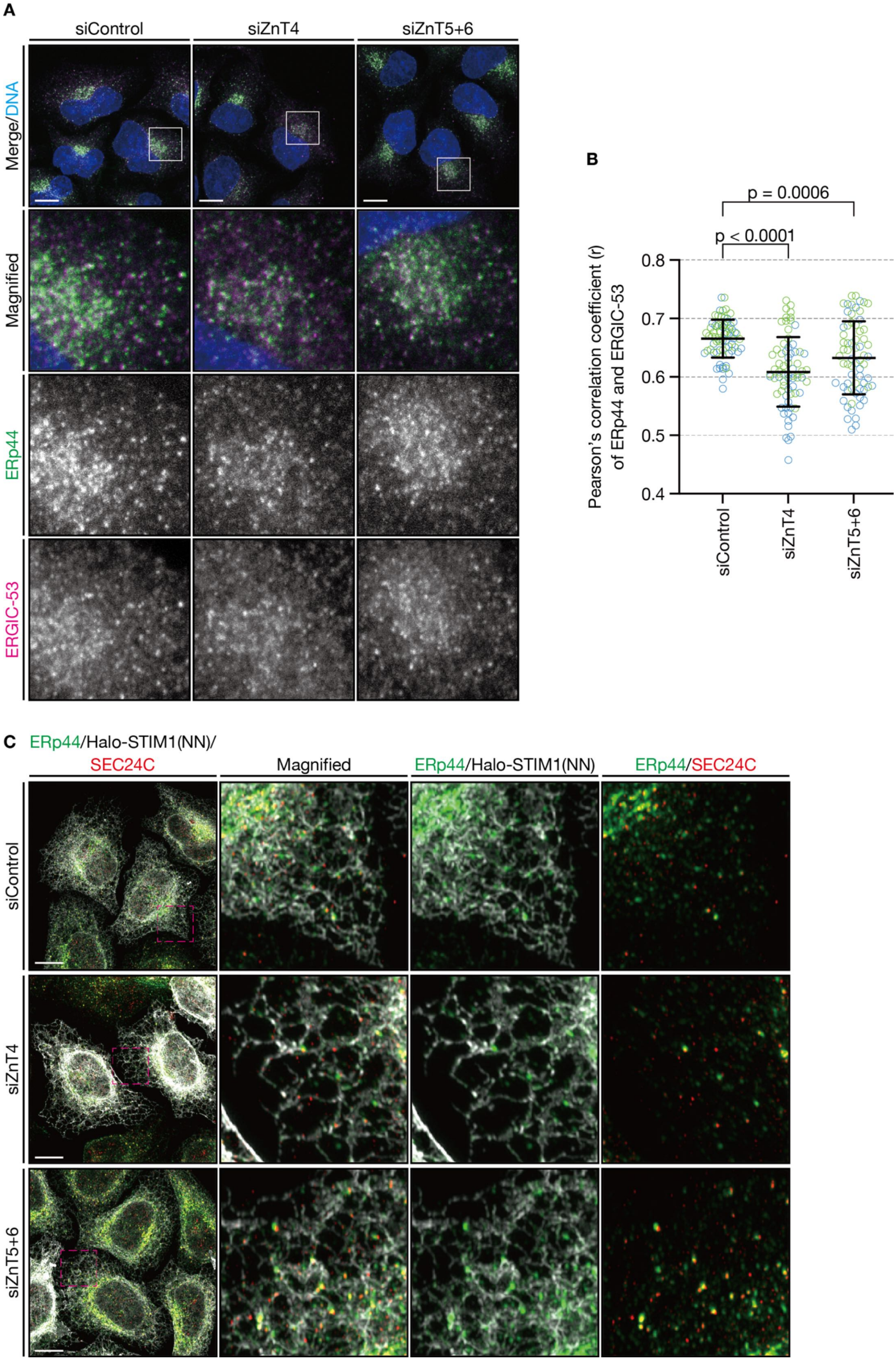

A
siControl
siZnT4
siZnT5+6
Merge/DNA
Magnified
ERp44
ERGIC-53
B
p = 0.0006
p < 0.0001
Pearson's correlation coefficient (r) of ERp44 and ERGIC-53
0.8
0.7
0.6
0.5
0.4
siControl
siZnT4
siZnT5+6
C
ERp44/Halo-STIM1(NN)/SEC24C
Magnified
ERp44/Halo-STIM1(NN)
ERp44/SEC24C
siControl
siZnT4
siZnT5+6

**Supplementary Figure 9. ZnT4- or ZnT5/6-knockdown relocated ERp44 to the ER**

(A) Representative fluorescence images of ERp44 (green) and ERGIC-53 (magenta) in HeLa Kyoto cells transfected with indicated siRNAs. Scale bars, 10 µm.

(B) Quantitative analysis of Pearson's correlation coefficients of the co-localization of endogenous ERp44 and ERGIC-53 based on the immunofluorescence images shown in (A). Circular dots indicate each datapoint, and bars indicate the means ± SD. siControl, n = 71 cells; siZnT4, n = 70 cells; siZnT5+6, n = 67 cells from 2 independent experiments. One-way ANOVA followed by Dunnett's multiple comparison test was used for statistical analysis.

(C) HeLa Kyoto cells were transfected with Halo-STIM1(NN), an ER marker, and indicated siRNAs. Cells were incubated with HTL-TMR to label the Halo-STIM1(NN) and then fixed with glyoxal solution. ERp44 and SEC24C, an ERES marker, were immunostained. Magnified images revealed most of ERp44 signals were associated with the ER and/or ERES. Scale bars, 10 µm. Images are representatives of two independent experiments.

Source numerical data are provided in the Source Data file.

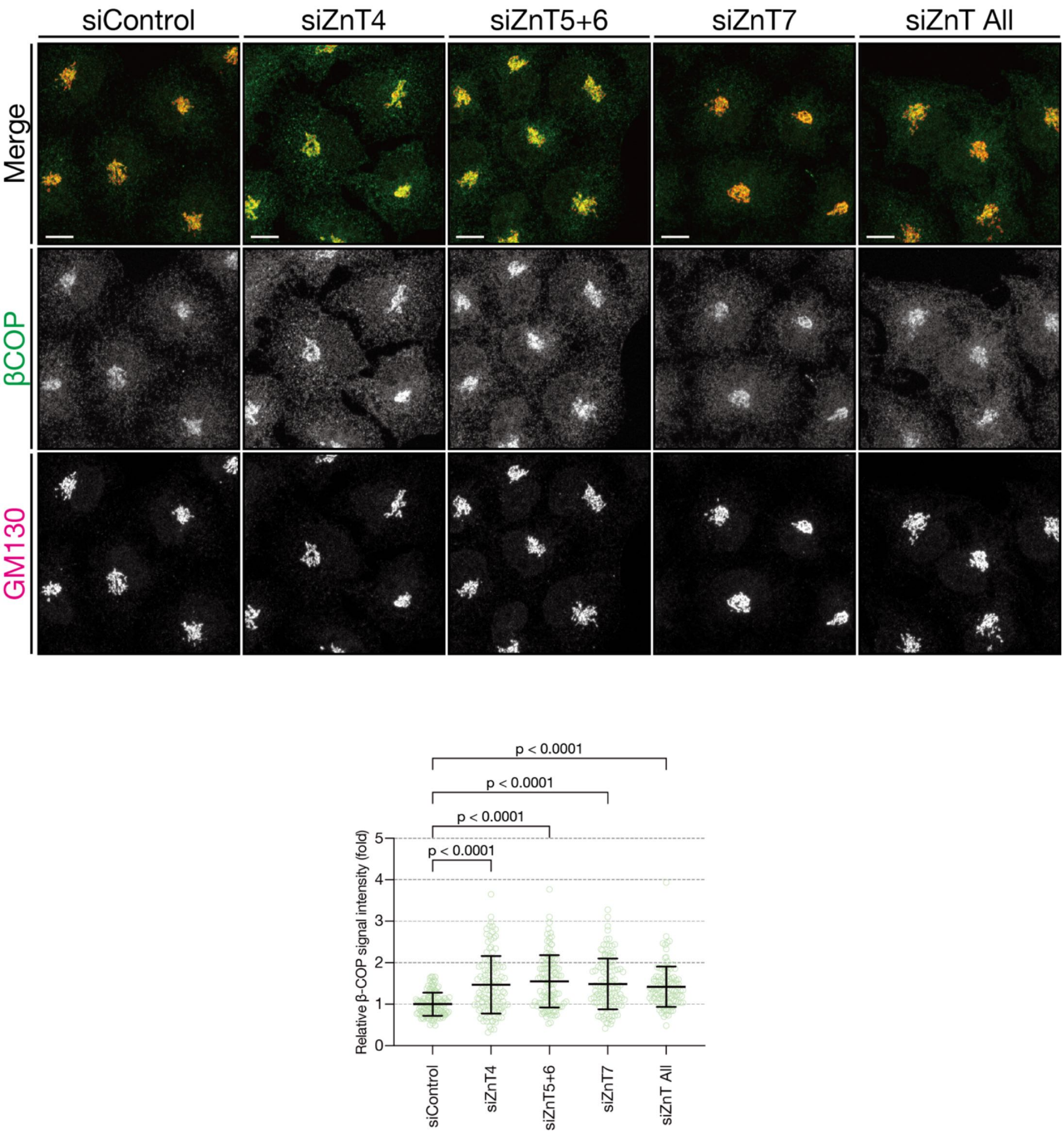


**Supplementary Figure 10. ZnTs-knockdown does not impair the recruitment of COPI coatomer to the Golgi apparatus**

HeLa Kyoto cells transfected with indicated siRNAs were fixed and immunostained for βCOP and GM130. Relative signal intensities of βCOP on the Golgi, determined by GM130 signals, were quantified and summarized. siControl, n = 127 cells; siZnT4, n = 119 cells; siZnT5+6, n = 116 cells; siZnT7, n = 115 cells; siZnT All, n = 111 cells from 2 independent experiments. Bars are the means ± SD. One-way ANOVA followed by Dunnett's multiple comparison test was used for statistical analysis. Scale bars, 10 μm. Source numerical data are provided in the Source Data file.

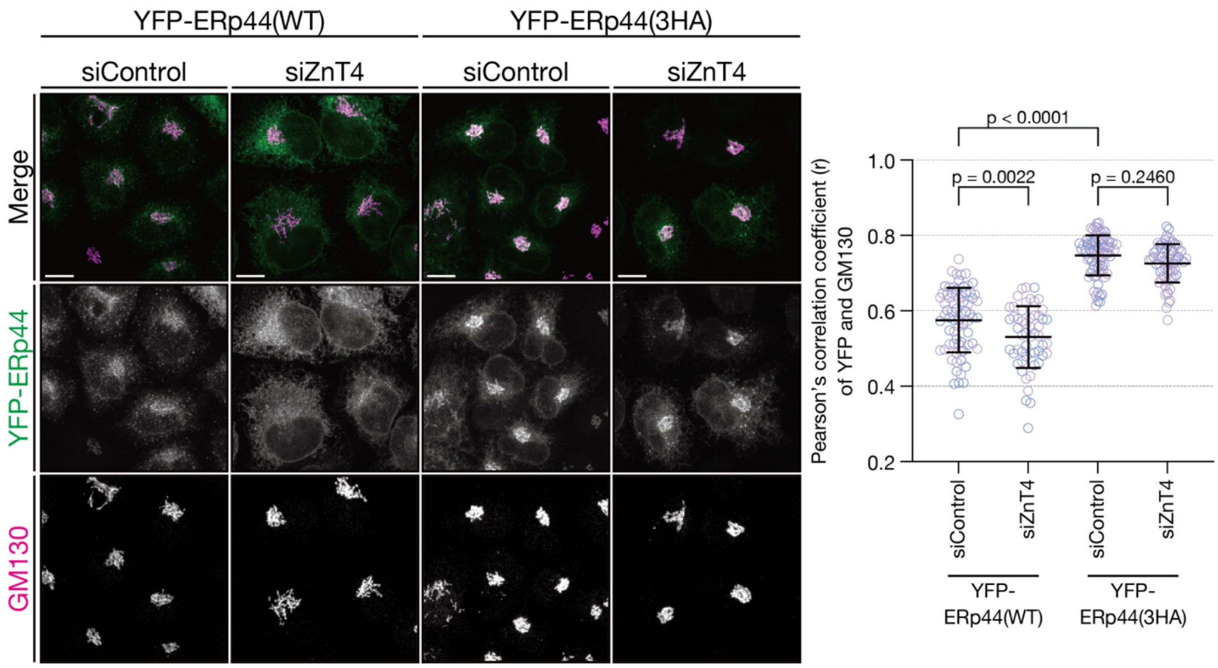


**Supplementary Figure 11. ZnT4-knockdown did not accelerate the Golgi to ER trafficking generally**

HeLa Kyoto cells were transfected with indicated siRNAs. After 12 h incubation, cells were further transfected with YFP-ERp44(WT) or YFP-ERp44(3HA). After additional 36 h incubation, cells were fixed and immunostained for GM130. Fluorescence images were acquired with laser scanning confocal microscopy equipped with 63x oil-immersion lens (NA = 1.40) and analyzed with Fiji. Pearson's correlation coefficient of YFP and GM130 revealed that ZnT4-silencing reduced the Golgi localization of YFP-ERp44(WT) but not YFP-ERp44(3HA), indicating that ZnT4-knockdown doesn't accelerate the general trafficking from the Golgi to the ER. YFP-ERp44(WT) + siControl, n = 72 cells; YFP-ERp44(WT) + siZnT4, n = 54 cells; YFP-ERp44(3HA) + siControl, n = 80 cells; YFP-ERp44(3HA) + siZnT4, n = 65 cells from 2 independent experiments. Circular dot indicates each datapoint. Bars are the means ± SD. One-way ANOVA followed by Tukey's multiple comparison test was used for statistical analysis. Scale bars, 10 µm.

Source numerical data are provided in the Source Data file.

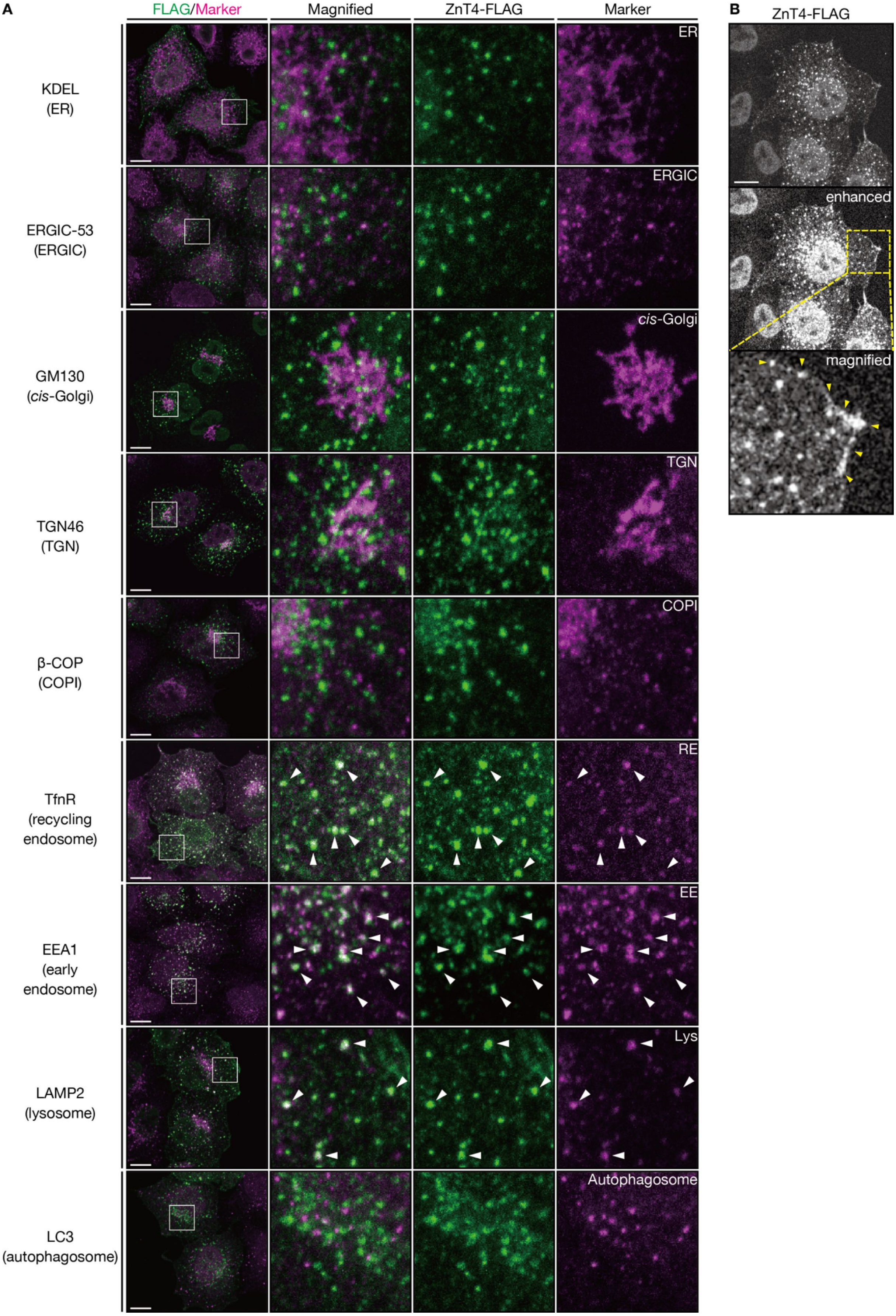

A
FLAG/Marker
Magnified
ZnT4-FLAG
Marker
KDEL
(ER)
ER
ERGIC-53
(ERGIC)
ERGIC
GM130
(cis-Golgi)
cis-Golgi
TGN46
(TGN)
TGN
β-COP
(COPI)
COPI
TfnR
(recycling
endosome)
RE
EEA1
(early
endosome)
EE
LAMP2
(lysosome)
Lys
LC3
(autophagosome)
Autophagosome
B
ZnT4-FLAG
enhanced
magnified

**Supplementary Figure 12. Localization of exogenously expressed ZnT4-FLAG**

(A) HeLa Kyoto cells were transfected with ZnT4-FLAG, fixed and coimmunostained for FLAG (green) with several organelle markers (magenta), including KDEL (ER), ERGIC-53 (ERGIC), GM130 (*cis*-Golgi), TGN46 (TGN), βCOP (COPI), transferrin receptor (TfnR; recycling endosome), EEA1 (early endosome), LAMP2 (lysosome), and LC3 (autophagosome). ZnT4-FLAG showed slight colocalization with GM130 and TGN46, but clear colocalization with TfnR, EEA1, and LAMP2, as indicated by arrowheads. Images are representatives of two independent experiments. Scale bars, 10 μm.

(B) A representative image of ZnT4-FLAG. Enhanced and magnified images show some portion of ZnT4-FLAG localize at the plasma membrane as indicated by yellow arrowheads. Images are representatives of two independent experiments. Scale bar, 10 μm.

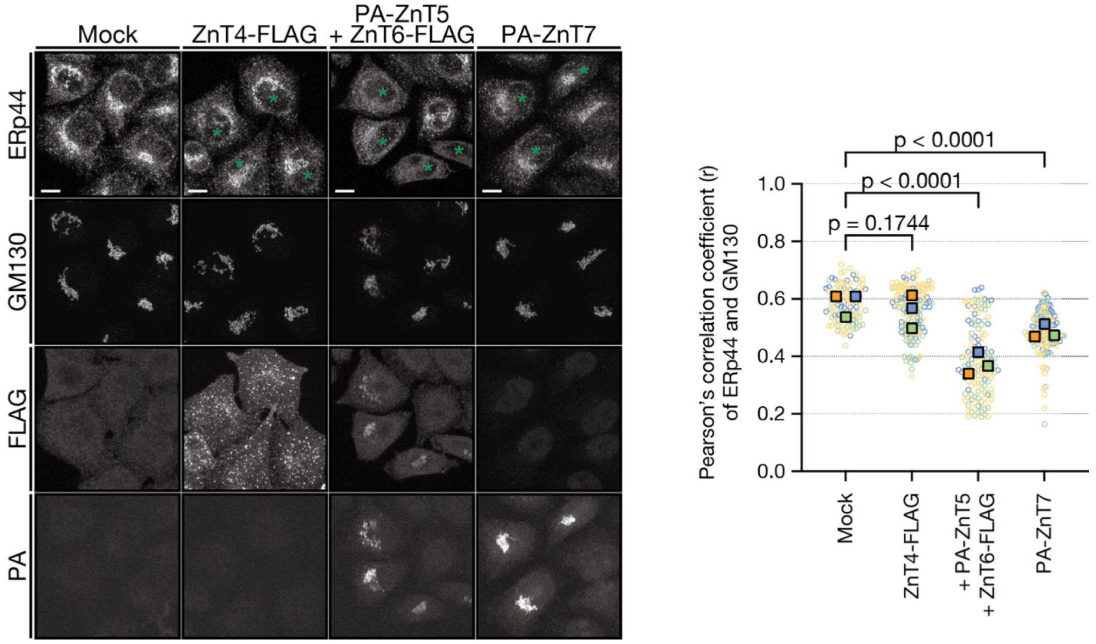


**Supplementary Figure 13. Effects of overexpression of ZnTs on the localization of ERp44**

HeLa Kyoto cells transfected with indicated ZnT-expressing plasmids were fixed and immunostained for ERp44, GM130, FLAG, and PA. Fluorescence images were acquired by a laser scanning confocal microscopy (Zeiss LSM980) equipped with 63x oil-immersion lens (NA = 1.40). Green asterisks indicate ZnT-expressing cells. Pearson's correlation coefficient of ERp44 and GM130 was analyzed with Fiji. Circular dots indicate individual data points, and rectangle dots indicate the median of each experiment (Mock, n = 47 cells; ZnT4-FLAG, n = 58 cells; PA-ZnT5 + ZnT6-FLAG, n = 43 cells; PA-ZnT7, n = 36). Data were analyzed by Dunnett's multiple comparison test (two-sided). Scale bars, 10 µm.

Source numerical data are provided in the Source Data file.

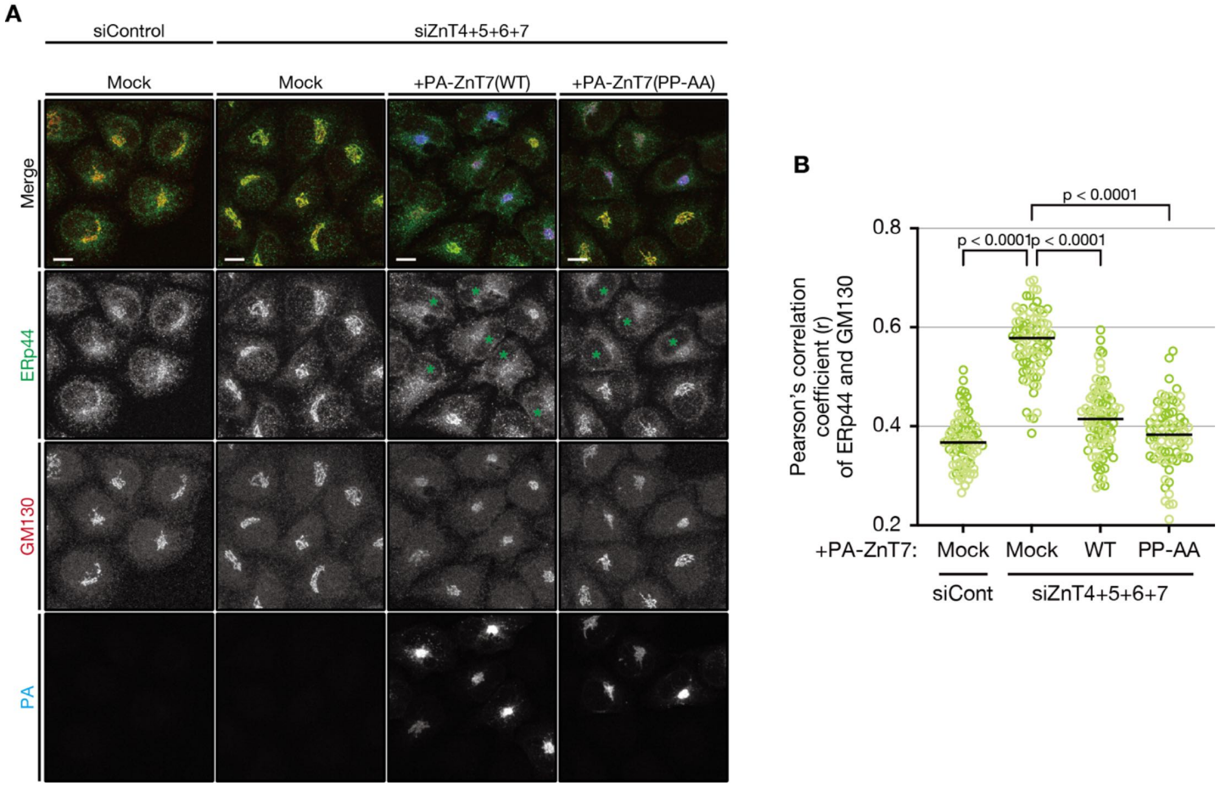


**Supplementary Figure 14. Di-proline motif of ZnT7 is dispensable for regulation of localization of ERp44 in the ESP**

(A) Immunofluorescence images showing the intracellular localization of ERp44, GM130 and PA-ZnT7. HeLa Kyoto cells were transfected with siRNAs against ZnT4/5/6/7 simultaneously or control siRNAs. After 24 h incubation, cells were further transfected with PA-ZnT7(WT) or (PP-AA) and cultured for an additional 24 h. Cells were fixed and immunostained for ERp44 (green), GM130 (red), and PA (blue). Green asterisks indicate PA-ZnT7-positive cells. Scale bars, 10 μm.

(B) Quantitative analysis of Pearson's correlation coefficients of the co-localization of ERp44 and GM130 based on the immunofluorescence images shown in (A). Only PA-positive cells were analyzed. Dots indicate individual datapoints (siCont + Mock, n = 82; siZnT4+5+6+7 + Mock, n = 82; siZnT4+5+6+7 + PA-ZnT7(WT), n = 83; siZnT4+5+6+7 + PA-ZnT7(PP-AA), n = 75 cells from 2 independent experiments). One-way ANOVA followed by Tukey's test was used for statistical analysis. Bars indicate the means.

Source numerical data are provided in the Source Data file.

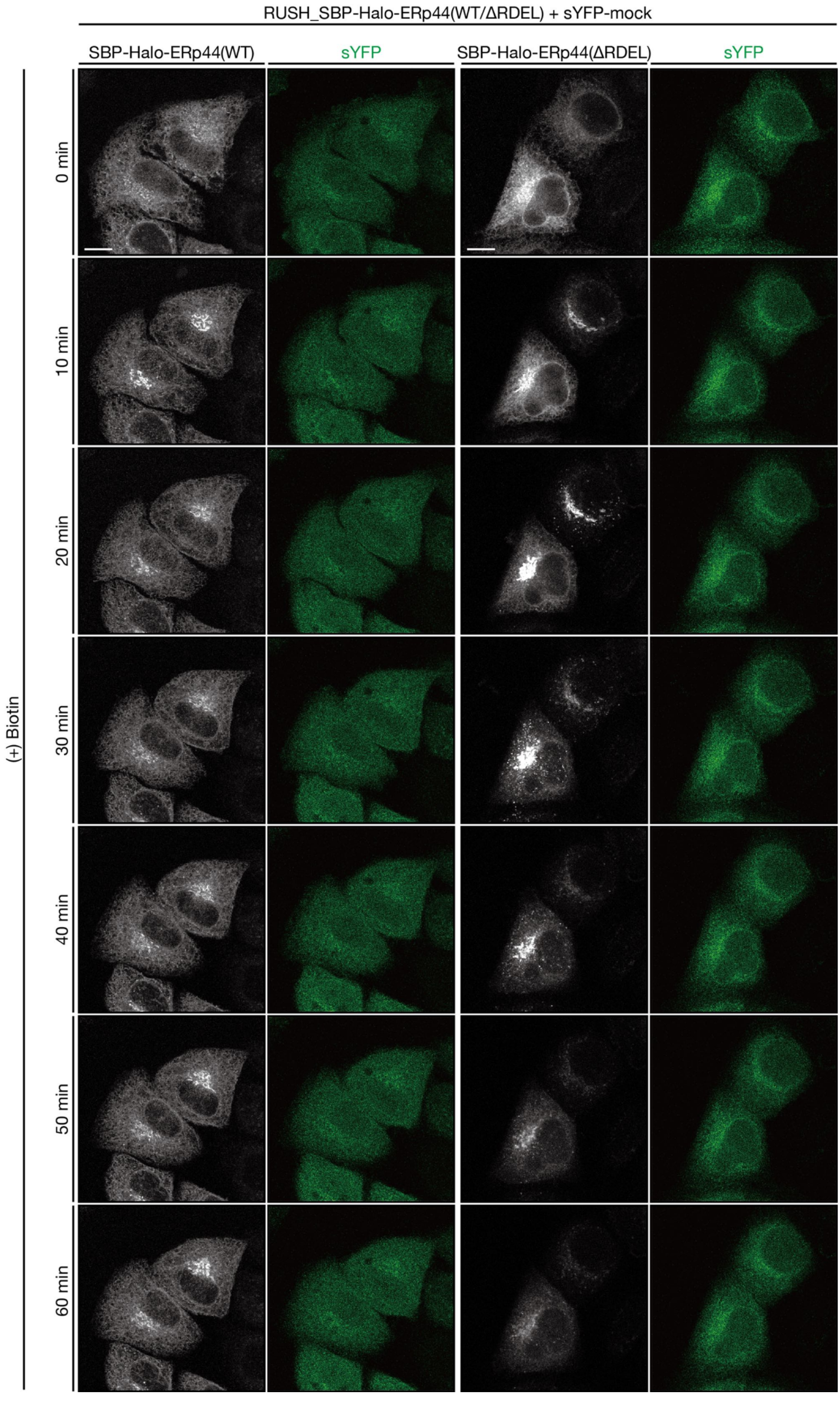

RUSH_SBP-Halo-ERp44(WT/ΔRDEL) + sYFP-mock
SBP-Halo-ERp44(WT)
sYFP
SBP-Halo-ERp44(ΔRDEL)
sYFP
(+) Biotin
0 min
10 min
20 min
30 min
40 min
50 min
60 min

**Supplementary Figure 15. Representative snapshots of the time-lapse imaging in the RUSH assay of SBP-Halo-ERp44(WT) or SBP-Halo-ERp44(ΔRDEL)**

HeLa Kyoto cells were co-transfected with signal sequence-YFP (sYFP) and pRUSH-SBP-Halo-ERp44(WT) or pRUSH-SBP-Halo-ERp44(ΔRDEL). Halo proteins were labeled with 5 nM of HTL-TMR and cells. The time-lapse imagings were carried out with Airyscan2 multiplex 4Y mode on LSM980 (Carl ZEISS) equipped with Plan Apochromat 40× lens (NA = 0.95). Fluorescence images were acquired every 1 min for 65 min. After 5 min of imaging, biotin solution was added into the culturing medium (final 80 μM), and the addition time is set as 0 min. At 0 min, both of SBP-Halo-ERp44(WT) and (ΔRDEL) showed a typical ER-like network structures in cells. However, while SBP-Halo-ERp44(WT) represented the ER-like network structure throughout the analysis, SBP-Halo-ERp44(ΔRDEL) lost the ER-like localization and entire signal intensity after the biotin treatment. Given that sYFP kept the signals, SBP-Halo-ERp44(ΔRDEL) should be secreted out of cells by biotin stimulation. These data also imply that SBP-ERp44(WT) is retrogradely transported from the Golgi to the ER in this experiment. Scale bars, 10 μm. See also Supplementary Movies 5 and 6.

A

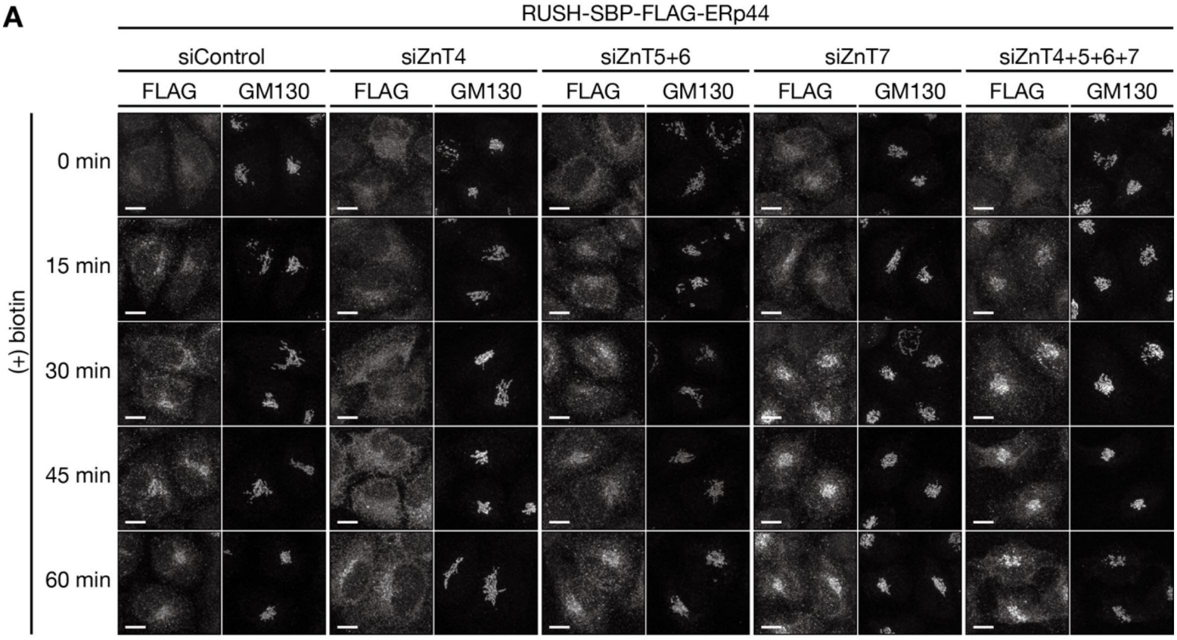
RUSH-SBP-FLAG-ERp44
siControl
siZnT4
siZnT5+6
siZnT7
siZnT4+5+6+7
FLAG
GM130
(+) biotin
0 min
15 min
30 min
45 min
60 min

B

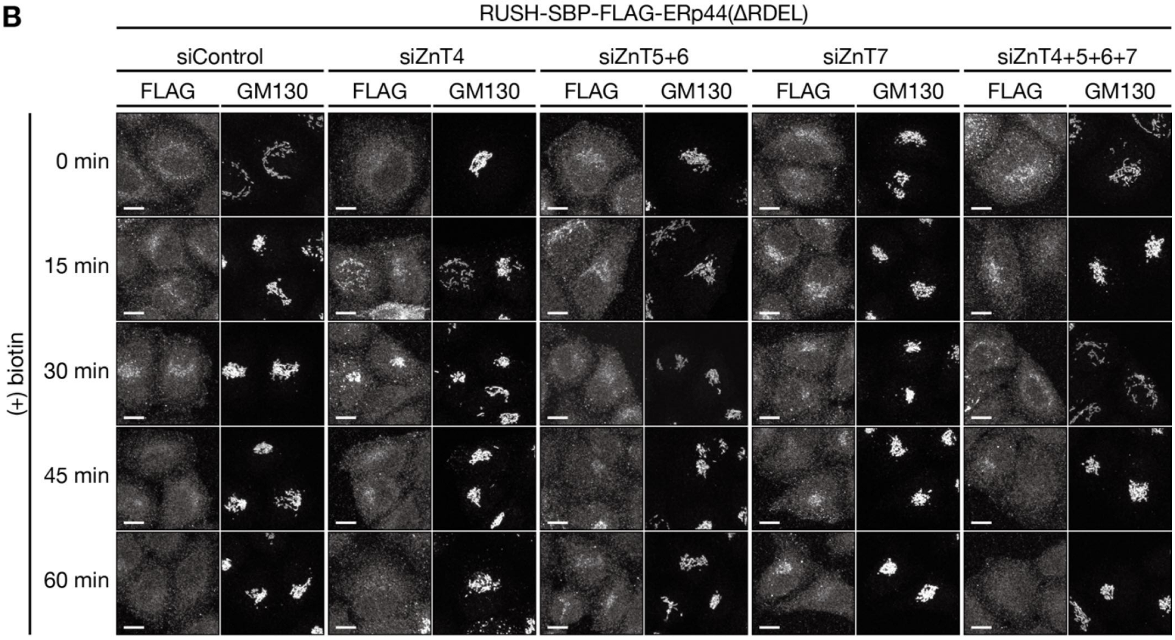
RUSH-SBP-FLAG-ERp44(ΔRDEL)
siControl
siZnT4
siZnT5+6
siZnT7
siZnT4+5+6+7
FLAG
GM130
(+) biotin
0 min
15 min
30 min
45 min
60 min

C

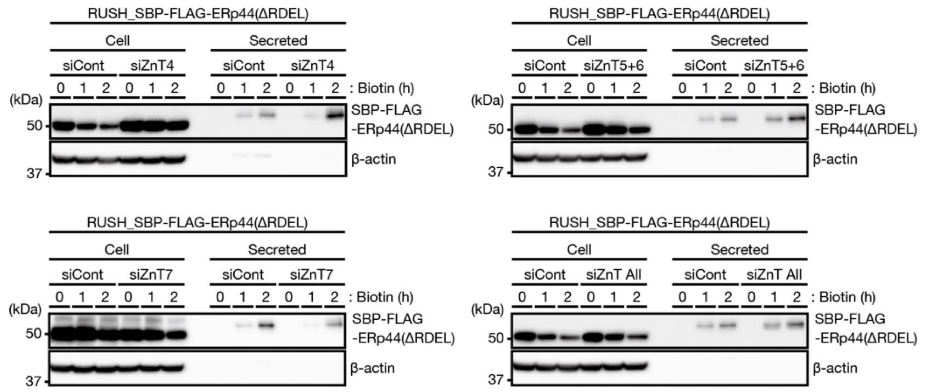
RUSH_SBP-FLAG-ERp44(ΔRDEL)
Cell
Secreted
siCont
siZnT4
siZnT5+6
siZnT7
siZnT All
0 1 2 0 1 2 0 1 2 0 1 2 : Biotin (h)
(kDa)
50
37
SBP-FLAG
-ERp44(ΔRDEL)
β-actin

**Supplementary Figure 16. Representative images of RUSH assays and secretion assay of RUSH-ERp44(ΔRDEL)**

(A) Representative images of RUSH-FLAG-ERp44 and GM130. Data are analyzed in Figure 6C. Scale bars, 10 μm.

(B) Representative images of RUSH-FLAG-ERp44(ΔRDEL) and GM130. Data are analyzed in Figure 6D. Scale bars, 10 μm.

(C) HeLa Kyoto cells transfected with indicated siRNAs were further transfected with RUSH-SBP-FLAG-ERp44(ΔRDEL), and then incubated for additional 36 h. Cells were washed with serum-free DMEM twice and incubated with DMEM with 10% streptavidin beads-treated FBS for 4 h. Culturing media were then exchanged to Opti-MEM with 40 μM biotin. After 0, 1, or 2 h incubation, conditioned media (CM) were harvested and precipitated by ice-cold 10% TCA. Cells were directly lysed in SDS-sample buffer (whole cell lysates, WCL). CM and WCL were analyzed by immunoblotting. Similar results were obtained from two independent experiments. Unprocessed blotting images are provided in the Source Data file.

**Supplementary Table 1. Sequences of siRNAs used for ZnTs knockdown**

| Name | Target Gene | siRNA ID | Sense sequence (5'-3') | Antisense sequence (5'-3') |
|---|---|---|---|---|
| siZnT4 #1 | SLC30A4 | S537495 | CAACCAAAAGAUUCACCUUTT | AAGGUGAAUCUUUUGGUUGGT |
| siZnT4 #2 | SLC30A4 | S15337 | CUAUCCAUAUGAACUAUGATT | UCAUAGUUCAUAUGGAUAGTT |
| siZnT5 #1 | SLC30A5 | S219 | CGAAUCAUAUGAUCUCCUATT | UAGGAGAUCAUAUGAUUCGAA |
| siZnT5 #2 | SLC30A5 | S220 | GCUCGAUUAACAAAAUAUATT | UAUAUUUUGUUAAUCGAGCGC |
| siZnT6 #1 | SLC30A6 | S31227 | CUUUUCAGAUCAUCACGUATT | UACGUGAUGAUCUGAAAAGTT |
| siZnT6 #2 | SLC30A6 | S31229 | CGAUGCUUUCUAUUCGGAATT | UUCCGAAUAGAAAGCAUCGTG |
| siZnT7 #1 | SLC30A7 | S45226 | GCUUAGGCUUGAUUUCCGATT | UCGGAAAUCAAGCCUAAGCAG |
| siZnT7 #2 | SLC30A7 | S45225 | CAGAUACACUUGGAAGUAUTT | AUACUUCCAAGUGUAUCUGCT |

**Supplementary Table 2. IDs of PrimePCR assay for qRT-PCR experiments**

| Gene | PrimePCR assay ID |
|---|---|
| SLC30A4 | qHsaCED0042990 |
| SLC30A5 | qHsaCED0005336 |
| SLC30A6 | qHsaCID0010138 |
| SLC30A7 | qHsaCID0016780 |
| PGK1 | qHsaCED0042912 |
| MT2A | qHsaCED0044076 |
| SLC30A1 | qHsaCED0046700 |
| SLC39A14 | qHsaCED0002314 |

**Supplementary Table 3. Primers used in this work**

| Name | Sequence (5'-3') |
|---|---|
| hB4GALT1_cloning1st_fw | CACACCCTTCTTAAAGCGG |
| hB4GALT1_cloning1st_rv | GACCGAGGTCAAGTTGCTAG |
| pEF4EcoRI_GalT(1-61)_Fw_Infu | CCAGTGTGGTGGAATTCGAATTCGCCACCATGAGGCTTCGGGAGCCG |
| hB4GALT1(1-61)_pEF4EcoRI_Rv_Infu | GCTGGATATCTGCAGAATTCCACTGCAGCGGTGTGGAGACTC |
| hTPST2_mRNA_Fw | CGCTAACCTGTCGCTGAAG |
| hTPST2_mRNA_Rv | GAGGTCCGATTTCCACTTAAATG |
| pEF4EcoRI_kozak_hTPST2_Fw_Infu | CCAGTGTGGTGGAATTCGAATTCGCCACCATGCGCCTGTCGGTGCG |
| hTPST2_pEF4EcoRI_Rv_Infu | GCTGGATATCTGCAGAATTCCACGAGCTTCCTAAGTGGGA |
| hManII mRNA fw | CACAGTGCGCTGTCTCCTTTG |
| hManII mRNA rv | TCCAAACTCCACCATCTGGATTG |
| pEF4EcoRI_ManII(1-117)InFu Fw | CAGTGTGGTGGAATTCGAATTCGCCACCATGAAGTTAAGCCGCCAGTTCAC |
| pEF4EcoRI_ManII(1-117)InFu Rv | GATATCTGCAGAATTCCACAGACAGTCTGCAGTGTCAACTGAG |
| pHluorin2_fw | ATGGTGAGCAAGGGCGAG |
| pHluorin2_rv | CTTGTACAGCTCGTCCATGCC |
| pH_pEF4_rv | CTCGCCCTTGCTCACCATGCGGCCGCCACTGTG |
| pH_pEF4_fw | GACGAGCTGTACAAGTAGGCTCGAGTCTAGAGGGCCCGCG |
| STIM1NN_SS_rv | CTCGCCCTTGCTCACGGCCCCCGAGCTGGTTCCTG |
| STIM1NN_pH_fw | GACGAGCTGTACAAGAACTCTGAGGAGTCCACTGCAGCAGAGTTTTG |
| ERGIC53_SS_rv | CTCGCCCTTGCTCACGCCCCGGACGAAGCGACCGAG |
| ERGIC53_pH_fw | GACGAGCTGTACAAGGGTACTGGCGGGGGTGGTTCCTGTACC |
| pMal_pHluorin2_Rv | CTCGCCCTTGCTCACGAATTCGGATCCGTCGACGATATC |
| pMal_pHluorin2_Fw | GACGAGCTGTACAAGTAGAAATAAAACGAAAGGCTCAGTCGAAAGAC |
| pHluorin2_fw | ATGGTGAGCAAGGGCGAG |
| pHluorin2_rv | CTTGTACAGCTCGTCCATGCC |

| | |
|---|---|
| pH_pEF4_rv | CTCGCCCTTGCTCACCATGCGGCCGCCACTGTG |
| pH_pEF4_fw | GACGAGCTGTACAAGTAGGCTCGAGTCTAGAGGGCCCGCG |
| hZnT4 cloning mRNA fw | GCACTGCCCTCGAGAACTG |
| hZnT4 cloning mRNA rv | GGACACAGCTGTCAGGGATTC |
| hZnT4 BamHI fw | CTGGATCCATGGCCGGCTCTGGCGC |
| hZnT4 NotI rv | ATGCGGCCGCTTAGGGACTAGAACTCTGACAATTTG |
| ZnT4_sirNo1_Fw | CAACTAAGAGGTTTACCTTTGGATTTCATCGC |
| ZnT4_sirNo1_Rv | AGGTAAACCTCTTAGTTGGTGATTTTGATGAT |
| sirZnT4v3_ins_fw | CAACTAAGAGGTTTACATTTGGATTTCATC |
| sirZnT4v3_ins_rv | CTCCATTTATTTCGTAATTCATGTGAATAG |
| sirZnT4v3_vec_fw | CTATTCACATGAATTACGAAATAAATGGAG |
| sirZnT4v3_vec_rv | GATGAAATCCAAATGTAAACCTCTTAGTTG |
| ZnT7(H70A)_QuickCh_Fw | CAGCGACAGCTTCGCCATGTTCTTCGACAG |
| ZnT7(H70A)_QuickCh_Rv | CTGTCGAAGAACATGGCGAAGCTGTCGCTG |
| ZnT7(D244A)_QuickCh_Fw | CACATTCTGGCCGCTACCCTGGGCAG |
| ZnT7(D244A)_QuickCh_Rv | CTGCCCAGGGTAGCGGCCAGAATGTG |
| ZnT7_PPAA_fw | TGGCCGCTGCTGATGTGCACCACGAGA |
| ZnT7_PPAA_rv | CATCAGCAGCGGCCAGGGCTCTTTCCAC |
| BamHI_kozak_ENPP1 Fw | GAGCTCGGATCCGCCACCATGGAGCGCGACGGCTGC |
| ENPP1_linker rv | CTCGTCCATGGAATTCCCGTCTTCTTGGCTAAAGGTTGGCAAATGTGTTTTC |
| linker_SBP fw | GGGAATTCCATGGACGAGAAG |
| SBP_EcoRI_FLAG rv | GTAATCTGCAGAATTCCATGGTTCACGTTGACCTTGTG |
| pEF4_EcoRI_FLAG fw | TGGAATTCTGCAGATTACAAGGATGAC |
| pEF4_BamHI_kozak rv | GGTGGCGGATCCGAGCTC |
| SmaI_IRES_rv | CTCCCGGGTTGTGGCAAG |
| XhoI_pIRESneo3_fw | CTCGAGGGCCAATTAATTAACTCTAGATAACTG |
| IRES_SmaI_ENPP1_fw | GCCACAACCCGGGAGATGGAGCGCGACGGCTGC |
| FLAG_pIRESneo3_rv | TAATTGGCCCTCGAGTTACTTATCGTCGTCATCCTTGTAATCTGCAGAATTC |
| 44ssSBP fw | GTAACAACTGAAATAATGGACGAGAAGACCACTGGTTG |

| | |
|---|---|
| XhoI FLAG ERp44 rv InFu | TGTTATTTCCTCGAGCTTATCGTCGTCATCCTTGTAATCTGC |
| hERp44_ssEI SBP rv | TATTTCAGTTGTTACAGGAGTAAAAACCCAAGTTAC |
| XhoI FLAG ERp44 fw InFu | CTCGAGGAAATAACAAGTCTTGATACAGAGAATATAGATGAAATTTTAAAC |
| IRES_SmaI_ERp44ss fw | GCCACAACCCGGGAGATGCATCCTGCCGTCTTC |
| pIRESneo_ERp44 rv | TAATTGGCCCTCGAGTTAAAGCTCATCTCGATCCCTC |
| pRUSH_KDEL Fw | AAAGATGAACTGTGACCGCGGCCGCATAGATAA |
| pRUSH_KDEL Rv | TCACAGTTCATCTTTCGGTCCGAGCTGCTGGAC |
| pRUSH_ERp44deltaRDEL Fw | GAGGGATTAACTCGAGGGCCAATTA |
| pRUSH_ERp44deltaRDEL Rv | TCGAGTTAATCCCTCAATAGAGTATA |
