# Supplementary material for: Zinc homeostasis governed by Golgi-resident ZnT family members regulates ERp44-mediated proteostasis at the ER-Golgi interface
Source: Nat Commun. 2023 May 9;14:2683. doi: 10.1038/s41467-023-38397-6 (PMC10170084; doi:10.1038/s41467-023-38397-6)
Supplement: Supplementary file 3 — Description of Additional Supplementary Files [file 41467_2023_38397_MOESM3_ESM.pdf]

Description of Additional Supplementary Files

File Name: Supplementary Movie 1
Description: 3D projection image of the Golgi mini-stack (X-axis rotation). ZnT6 (green), Halo-ERGIC-53 (red), and GM130 (blue) are indicated, related to Fig. 2a (upper panel).

File Name: Supplementary Movie 2
Description: 3D projection image of the Golgi mini-stack (X-axis rotation). ZnT7 (green), Halo-ERGIC-53 (red), and GM130 (blue) are indicated, related to Fig. 2a (lower panel).

File Name: Supplementary Movie 3
Description: 3D projection image of the Golgi mini-stack (X-axis rotation). ZnT6 (green), TPST2-Halo (red), and GM130 (blue) are indicated, related to Fig. 2c (upper panel).

File Name: Supplementary Movie 4
Description: 3D projection image of the Golgi mini-stack (X-axis rotation). ZnT7 (green), TPST2-Halo (red), and GM130 (blue) are indicated, related to Fig. 2c (lower panel).

File Name: Supplementary Movie 5
Description: Fluorescence time-lapse images of HeLa Kyoto cells co-transfected with signal sequence-YFP (sYFP) and pRUSH-SBP-Halo-ERp44(WT). Cells were treated with biotin at T = 0 min, related to Supplementary Fig. 15 (left panel).

File Name: Supplementary Movie 6
Description: Fluorescence time-lapse images of HeLa Kyoto cells co-transfected with signal sequence-YFP (sYFP) and pRUSH-SBP-Halo-ERp44(ΔRDEL). Cells were treated with biotin at T = 0 min, related to Supplementary Fig. 15 (right panel).

File Name: Supplementary Movie 7
Description: Fluorescence time-lapse images of HeLa Kyoto cells transfected with siControl. Cells were further co-transfected with ManII-pHluorin2 and pRUSH-SBP-

Halo-ERp44(WT). Cells were treated with biotin at T = 0 min, related to Fig. 7f (upper panel).

File Name: Supplementary Movie 8
Description: Fluorescence time-lapse images of HeLa Kyoto cells transfected with siZnT4. Cells were further co-transfected with ManII-pHluorin2 and pRUSH-SBP-Halo-ERp44(WT). Cells were treated with biotin at T = 0 min, related to Fig. 7f (lower panel).
