# Supplementary material for: Zinc homeostasis governed by Golgi-resident ZnT family members regulates ERp44-mediated proteostasis at the ER-Golgi interface
Source: Nat Commun. 2023 May 9;14:2683. doi: 10.1038/s41467-023-38397-6 (PMC10170084; doi:10.1038/s41467-023-38397-6)
Supplement: Supplementary file 12 — Reporting Summary [file 41467_2023_38397_MOESM12_ESM.pdf]

nature portfolio

Corresponding author(s): Kenji Inaba

Last updated by author(s): Apr 11, 2023

Reporting Summary

Nature Portfolio wishes to improve the reproducibility of the work that we publish. This form provides structure for consistency and transparency in reporting. For further information on Nature Portfolio policies, see our Editorial Policies and the Editorial Policy Checklist.

Statistics

For all statistical analyses, confirm that the following items are present in the figure legend, table legend, main text, or Methods section.

n/a	Confirmed	
☐	☒	The exact sample size (*n*) for each experimental group/condition, given as a discrete number and unit of measurement
☐	☒	A statement on whether measurements were taken from distinct samples or whether the same sample was measured repeatedly
☐	☒	The statistical test(s) used AND whether they are one- or two-sided<br>*Only common tests should be described solely by name; describe more complex techniques in the Methods section.*
☒	☐	A description of all covariates tested
☐	☒	A description of any assumptions or corrections, such as tests of normality and adjustment for multiple comparisons
☐	☒	A full description of the statistical parameters including central tendency (e.g. means) or other basic estimates (e.g. regression coefficient) AND variation (e.g. standard deviation) or associated estimates of uncertainty (e.g. confidence intervals)
☐	☒	For null hypothesis testing, the test statistic (e.g. *F*, *t*, *r*) with confidence intervals, effect sizes, degrees of freedom and *P* value noted<br>*Give P values as exact values whenever suitable.*
☒	☐	For Bayesian analysis, information on the choice of priors and Markov chain Monte Carlo settings
☒	☐	For hierarchical and complex designs, identification of the appropriate level for tests and full reporting of outcomes
☐	☒	Estimates of effect sizes (e.g. Cohen's *d*, Pearson's *r*), indicating how they were calculated

Our web collection on statistics for biologists contains articles on many of the points above.

Software and code

Policy information about availability of computer code

Data collection	Fluorescence microscopy images were acquired using FluoView software ver 4.2 (Olympus) or ZEN software ver 3.4.91.00000 (ZEISS).
Data analysis	We used the following softwares for data analysis.<br>Fiji ver1.0 or ver 2.9.0 (NIH)<br>Image Lab ver5.2 (Bio-Rad)<br>GraphPad Prism ver7 or ver9.2.0/9.3.0 (GraphPad)<br>Chromagnon (Matsuda et al., 2018, Mol Biol Cell)<br>CFX Manager 3.1 (Bio-Rad)

For manuscripts utilizing custom algorithms or software that are central to the research but not yet described in published literature, software must be made available to editors and reviewers. We strongly encourage code deposition in a community repository (e.g. GitHub). See the Nature Portfolio guidelines for submitting code & software for further information.

Data

Policy information about availability of data

All manuscripts must include a data availability statement. This statement should provide the following information, where applicable:

- Accession codes, unique identifiers, or web links for publicly available datasets
- A description of any restrictions on data availability
- For clinical datasets or third party data, please ensure that the statement adheres to our policy

The datasets generated during and/or analyzed during the current study are available within the supplementary information. Source data are provided with this paper. All other data supporting the conclusions and findings of this study are available from the corresponding author on reasonable request.

Field-specific reporting

Please select the one below that is the best fit for your research. If you are not sure, read the appropriate sections before making your selection.

☒ Life sciences ☐ Behavioural & social sciences ☐ Ecological, evolutionary & environmental sciences

For a reference copy of the document with all sections, see nature.com/documents/nr-reporting-summary-flat.pdf

Life sciences study design

All studies must disclose on these points even when the disclosure is negative.

Sample size

No statistical methods were used to predetermine sample size. The sample size was chosen based on our previous experiences in similar experiments. More detailed explanations are as below.

Fig 1d: Experiments were repeated 3 or 4 times, and all data points were considered in the statistical analyses. Fig 2: Experiments were repeated 3 times, and randomly chosen ministacks were subjected to the analyses. Figs 3, 4, S9B, S10, S11, S13, S14: more than 60 cells from 2 or 3 independent experiments were analyzed, which is enough to yield power to detect specific effects based on our previous experience (PMID: 30723194). Fig5: Experiments were repeated 3–5 times and all results were subjected to the analyses. Fig6: more than 50 cells from 2 independent experiments were analyzed to obtain the reliable mean value of each condition. Fig7: Experiments were repeated twice and all data points were considered in the statistical analyses. FigS2F, S2H: Experiments were repeated 3 times and all data points were considered in the statistical analyses. FigS3A: Experiments were repeated 3 times and all data were subjected to the analyses. FigS5: Experiments were repeated twice, and all data points were subjected to the analyses. FigS6A: Experiments were repeated 4 times to obtain reliable values of each condition. FigS6: Experiments were repeated 3 times, and all data points were considered in the statistical analyses.

Data exclusions

Cells that were only partially seen around the periphery of the microscopy imaging field were excluded from the analysis. In live-cell imagings, cells that died during the observation were excluded from the analysis.

Replication

All experiments were repeated at least twice with similar results.

Randomization

Randomization is not relevant to this work since we have not allocated any samples into experimental groups.

Blinding

Blinding was not relevant to this work since we have not done group allocation in any experiments.

Reporting for specific materials, systems and methods

We require information from authors about some types of materials, experimental systems and methods used in many studies. Here, indicate whether each material, system or method listed is relevant to your study. If you are not sure if a list item applies to your research, read the appropriate section before selecting a response.

Materials & experimental systems

n/a	Involved in the study	
☐	☒	Antibodies
☐	☒	Eukaryotic cell lines
☒	☐	Palaeontology and archaeology
☒	☐	Animals and other organisms
☒	☐	Human research participants
☒	☐	Clinical data
☒	☐	Dual use research of concern

Methods

n/a	Involved in the study	
☒	☐	ChIP-seq
☒	☐	Flow cytometry
☒	☐	MRI-based neuroimaging

Antibodies

Antibodies used

Mouse monoclonal antibodies; Anti-beta-actin (1:5000 for Western blotting, SIGMA, A1978, clone AC-15, lot# 029M4883V), Anti-DYKDDDDK tag (1:200 for Immunoprecipitation, Fujifilm Wako Pure Chemicals, clone 1E6, lot# WDP4261), Anti-ERp44 (1:1000 for Western blotting, house-made, clone 36C9), Anti-ERp44 (1:50 for Immunofluorescence, house-made, clone 2D5), Anti-FLAG-Peroxidase (1:10000 for Western blotting, SIGMA, A8592, clone M2), Anti-FLAG (1:2000 for Immunofluorescence, SIGMA, F1804, clone M2), Anti-GAPDH-Peroxidase (1:20000 for Western blotting, SIGMA, G9295, clone GAPDH-71.1, lot# 029M4800V), Anti-GM130 (1:2000 for Immunofluorescence, BD biosciences, 610822, clone 35/GM130, lot# 7069938), Anti-golgin97 (1:200 for Immunofluorescence, Invitrogen, A-21270, clone CDF4)
Rat monoclonal antibodies; Anti-PA tag (1:10000 for Western blotting, 1:5000 for Immunofluorescence, Fujifilm Wako Pure Chemicals, clone NZ-1, lot# CAQ4140)
Rabbit monoclonal antibody; Anti-ERp44 (1:1000 for Western blotting, CST, 3798, clone D17A6, lot# 2)
Rabbit polyclonal antibody; Anti-mannnosidase II (1:200 for Immunofluorescence, SIGMA, AB3712, lot# 3819983), Anti-giantin (1:2000 for Immunofluorescence, Biolegend, 924302, lot# B233848), Anti-GM130 (1:2000 for Immunofluorescence, MBL, PM061, Lot# 004), Anti-ZnT6 (1:500 for Immunofluorescence, 1:1000 for Western blotting, Atlas antibodies, HPA057328, Lot# R80549), Anti-

ZnT7 (1:200 for Immunofluorescence, 1:500 for Western blotting, this study), Anti-SEC24C (1:500 for Immunofluorescence, SIGMA, HPA040196, Lot# 107244), Anti-beta COP (1:1000 for Immunofluorescence, Abcam, ab2899), Anti-DDDDK (1:4000 for immunofluorescence, MBL, PM020, Lot# 028).
Fluorescence dye conjugated antibody; Anti-Mouse-IgG-CF488A (1:2000 for Immunofluorescence, biotium, 20014-1, Lot# 17C0125), Anti-Mouse-IgG-CF568 (1:2000 for Immunofluorescence, biotium, 20101-1, Lot# 16C0825), Anti-Mouse-IgG-CF633 (1:2000 for Immunofluorescence, biotium, 20121-1, Lot# 12C0106), Anti-Rabbit-IgG-CF488 (1:2000 for Immunofluorescence, biotium, 20015-1, Lot# 19C1009), Anti-Rabbit-IgG-CF568 (1:2000 for Immunofluorescence, biotium, 20103-1, Lot# 16C0422).

Validation

Mouse monoclonal antibodies:
Anti-beta-actin; supplier (https://www.sigmaaldrich.com/JP/ja/product/sigma/a5441)
Anti-DYKDDDDK tag; supplier (https://labchem-wako.fujifilm.com/jp/product/detail/W01W0101-2278.html)
Anti-ERp44 (clone 36C9, 2D5); Anelli T. et al., EMBO J., 26, 4177-4188, 2007
Anti-FLAG-Peroxidase; supplier (https://www.sigmaaldrich.com/catalog/product/sigma/f1804?lang=ja&region=JP)
Anti-GAPDH-Peroxidase; supplier (https://www.sigmaaldrich.com/JP/ja/product/sigma/g9295)
Anti-GM130; supplier (https://www.bdbiosciences.com/en-nz/products/reagents/microscopy-imaging-reagents/immunofluorescence-reagents/purified-mouse-anti-gm130.610822)
Anti-HaloTag; supplier (https://www.promega.jp/products/protein-detection/primary-and-secondary-antibodies/anti-halotag-monoclonal-antibody/?catNum=G9211#specifications)
Anti-golgin97; supplier (https://www.thermofisher.com/antibody/product/Golgin-97-Antibody-clone-CDF4-Monoclonal/14-9767-82)
Rat monoclonal antibodies:
Anti-DYKDDDDK tag; supplier (https://labchem-wako.fujifilm.com/jp/product/detail/W01W0101-2362.html)
Anti-PA tag; supplier (https://labchem-wako.fujifilm.com/jp/product/detail/W01W0101-2586.html)
Rabbit monoclonal antibodies:
Anti-ERp44; supplier (https://www.cellsignal.com/products/primary-antibodies/erp44-d17a6-xp-rabbit-mab/3798)
Rabbit polyclonal antibodies:
Anti-giantin; CiteAb (https://www.citeab.com/antibodies/2864094-924302-anti-giantin-antibody)
Anti-GM130; supplier (https://ruo.mbl.co.jp/bio/dtl/A/index.html?pcd=PM061)
Anti-ZnT6; this study (Supplementary Figure 7)
Anti-ZnT7; this study (Supplementary Figure 7)
Anti-Mannosidase II; supplier (https://www.sigmaaldrich.com/JP/ja/product/mm/ab3712)
Anti-SEC24C; supplier (https://www.sigmaaldrich.com/JP/ja/product/sigma/hpa040196)
Anti-beta COP; supplier (https://www.abcam.co.jp/products/primary-antibodies/beta-cop-antibody-ab2899.html)
Anti-DDDDK; supplier (https://ruo.mbl.co.jp/bio/dtl/A/?pcd=PM020)
Fluorescence dye conjugated antibodies; supplier (https://biotium.com/technology/immunofluorescence-microscopy/)

Eukaryotic cell lines

Policy information about cell lines

Cell line source(s)

HeLa Kyoto cell was established by Prof. Shu Narumiya (Kyoto Univ., Japan), and we received the cell line through Prof. Toru Hirota (JFCR, Japan) and Prof. Kozo Tanaka (Tohoku Univ., Japan). HEK293T cell was purchased from RIKEN BRC (RCB2202).

Authentication

None of the cell lines used have been authenticated.

Mycoplasma contamination

The cell line was tested for mycoplasma contamination using MycoStrip (InvivoGen) and confirmed to be negative.

Commonly misidentified lines
(See ICLAC register)

No commonly misidentified lines were used in this study.
